# Xylazine-Involved Fatal and Nonfatal Drug Overdoses in Tennessee From 2019 to 2022

**DOI:** 10.1001/jamanetworkopen.2023.24001

**Published:** 2023-07-18

**Authors:** Jessica Korona-Bailey, Edward Onyango, Kristi Frances Hall, Joshua Jayasundara, Sutapa Mukhopadhyay

**Affiliations:** 1 Office of Informatics and Analytics, Tennessee Department of Health, Nashville

## Abstract

This cross-sectional study examines the characteristics of overdoses and the potential association between drug seizures and fatal overdoses.

## Introduction

Xylazine is a veterinary drug that is traditionally used as a sedative and not listed as a controlled substance in the US. Research suggests that in humans xylazine may potentially increase fatal respiratory depression when used in combination with opioids.^1^ The Drug Enforcement Administration has seized xylazine in 48 states, and approximately 23% of fentanyl powder and 7% of fentanyl pills seized have contained xylazine.^2^ The Biden-Harris Administration designated the xylazine-fentanyl combination as an emerging threat.^3^ Given the increasing use of xylazine as an adulterant and its presence in Tennessee, we sought to determine the characteristics of xylazine-involved overdoses to inform prevention and outreach efforts.

## Methods

Fatal xylazine-involved overdoses were identified through searching for *xylazine* on death certificates and in toxicological results using Tennessee State Unintentional Drug Overdose Reporting System data from January 1, 2019, to December 31, 2022. A subanalysis of circumstances of deaths from January 1, 2019, to June 30, 2022, was conducted using autopsy narratives. The Tennessee Department of Health Institutional Review Board deemed this cross-sectional study exempt from ethics review and waived the informed consent requirement because it was considered as surveillance activity. We followed the STROBE reporting guideline.

Nonfatal overdoses that were suggestive of xylazine involvement during the study period were identified using the syndromic Electronic Surveillance System for the Early Notification of Community Based Epidemics by searching for the following terms in the chief concerns or discharge diagnosis field: *xylazine*, *rompun*, *anased*, *sedazine*, *tranq*, *tranq dope*, *sleep-cut*, *philly dope*, *cow tranquilizer*, and *horse tranquilizer*. Descriptive statistics were calculated. Race and ethnicity data were self-reported and analyzed to identify race-based patterns in substance use.

Patterns of fatal xylazine-involved overdoses from Tennessee death statistics file and drug seizures from the National Forensic Laboratory Information System^4^ were graphed. Xylazine concentration levels were scraped from toxicological files using R 4.2.2 with pdftools package 3.3.3 (R Foundation for Statistical Computing).

## Results

During the study period, 324 fatal xylazine-involved overdoses occurred in Tennessee in 210 males (64.8%) and 114 females (35.2%), with a mean (SD) age of 39.0 (11.0) years and predominately White race (246 [75.9%]). Bystanders were present in 87 cases (31.1%) and naloxone was administered in 96 cases (34.4%), yet only 5 decedents (1.8%) had a pulse on emergency medical services (EMS) arrival. Fentanyl was present in all deaths. Median (range) concentration of xylazine was 16 (5-610) ng/mL for femoral and iliac blood samples (Table).

**Table.** Characteristics of Xylazine-Involved Fatal Drug Overdoses in Tennessee^a^

Characteristic	No. (%)
**Xylazine-involved fatal overdoses**	
Total No. of overdoses	324
Age group, y	
18-24	23 (7.1)
25-34	111 (34.3)
35-44	95 (29.3)
45-54	58 (17.9)
≥55	36 (11.1)
Sex	
Female	114 (35.2)
Male	210 (64.8)
Race and ethnicity^b^	
Hispanic	9 (2.8)
Non-Hispanic Black	65 (20.1)
Non-Hispanic White	246 (75.9)
Grand region of death	
West	41 (12.7)
Middle	133 (41.1)
East	150 (46.3)
Death county	
Rural	43 (13.3)
Urban	281 (86.7)
**Subanalysis of deaths** ^c^	
Total No. of deaths	280
Route of administration	
Injection evidence	75 (26.7)
Snorting	23 (8.2)
Smoking	11 (3.9)
Ingestion	16 (5.7)
Overdose response	
Presence of bystander	87 (31.1)
Administration of naloxone	96 (34.3)
Presence of pulse	5 (1.8)
Death location	
Home	114 (40.7)
ED or outpatient	58 (20.7)
Other residence	26 (9.3)
Hotel or motel	22 (7.9)
Hospital inpatient	18 (6.4)
DOA	10 (3.6)
Other^d^	32 (11.4)
**Toxicological results^e^**	
Drugs found	
Opioids	280 (100)
Stimulants	151 (53.9)
Benzodiazepines	69 (24.6)
Alcohol	51 (18.2)
Cannabis	82 (29.3)
Individual substances found	
Fentanyl	280 (100)
Prescription opioids	47 (16.8)
Methamphetamines	109 (38.9)
Cocaine	42 (15.0)
Heroin	29 (10.4)
Xylazine concentration, median (range), ng/mL^f^	16 (5-610)

Abbreviations: DOA, dead on arrival; ED, emergency department.

^a^ Data from Tennessee State Unintentional Drug Overdose Reporting System from January 1, 2019, to December 31, 2022. Data from July 1 to December 31, 2022, were provisional. Nonfatal xylazine-involved overdose data are not shown due to small sample size.

^b^ Race and ethnicity were self-reported by a family member for the death certificate.

^c^ Data from January 1, 2019, to June 30, 2022.

^d^ Other included motor vehicles, parking lots, homeless camps, homeless shelters, and outdoor areas.

^e^ Substances were not mutually exclusive.

^f^ Median (range) concentration levels were for a subset of 228 cases with femoral or iliac blood samples that were scraped from toxicological files.

There were 14 nonfatal overdoses (11 males [78.6%], 3 females [21.4%]; mean [range] age, 39 [18-57] years). Fatal overdoses and drug seizures followed similar patterns. Before 2019, xylazine’s impact was low. However, from the second half of 2019 through the first half of 2022, fatal overdoses and drug seizures increased (352% and 1836%, respectively) (Figure).

**Figure.**
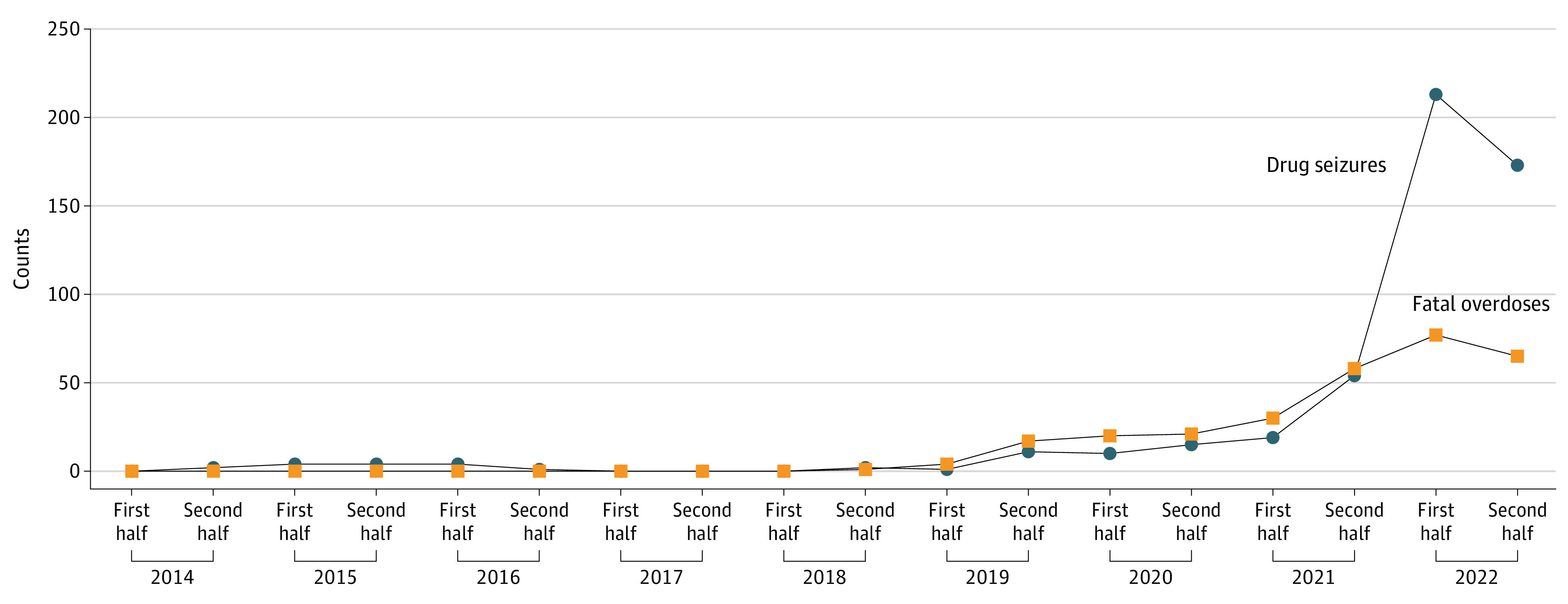
Patterns in Fatal Xylazine-Involved Overdoses and Drug Seizures in Tennessee From 2014 to 2022

## Discussion

Characteristics of fatal and nonfatal xylazine-involved overdoses in Tennessee were similar and followed patterns found in other states.^5^ However, the low number of nonfatal vs fatal overdoses was an important finding. Naloxone administration suggested that bystanders were equipped to respond to overdoses, yet the low proportion of individuals with a pulse on EMS arrival was concerning. Naloxone appeared to be not effective against xylazine, creating a need for swift medical intervention.

In many states, xylazine is scheduled to limit nonmedical use and naloxone administration is recommended for harm reduction.^2^ Additional strategies include point-of-use testing at syringe service programs and messaging campaigns warning against substance use when alone. Potential association between drug seizures and fatal overdoses may indicate future overdoses. Given xylazine’s rapid emergence, overdose prevention and data-driven policy advocacy should be research priorities to help decrease its use in communities.

A study limitation was the challenge of identifying nonfatal overdoses due to fast metabolism of xylazine and lack of testing.^6^ Future efforts to create definitions for xylazine-involved nonfatal overdoses and enhanced toxicological testing are warranted. Additionally, fatal circumstantial data were limited to those recorded in autopsy reports, potentially accounting for the low evidence of injection.

## Notes

Supplement.
Data Sharing Statement
