# Supplementary material for: Xylazine-Involved Fatal and Nonfatal Drug Overdoses in Tennessee From 2019 to 2022
Source: JAMA Netw Open. 2023 Jul 18;6(7):e2324001. doi: 10.1001/jamanetworkopen.2023.24001 (PMC10354674; doi:10.1001/jamanetworkopen.2023.24001)
Supplement: Supplement. — Data Sharing Statement [file jamanetwopen-e2324001-s001.pdf]

Data Sharing Statement

Korona-Bailey. Xylazine-Involved Fatal and Nonfatal Drug Overdoses in Tennessee From 2019 to 2022. *JAMA Netw Open*. Published July 18, 2023.
doi:10.1001/jamanetworkopen.2023.24001

Data
Data available: No

Additional Information
Explanation for why data not available: The data that support the findings are available upon request from the Tennessee Department of Health.
